# Cellular aspects of immunity involved in the development of atherosclerosis

**DOI:** 10.3389/fimmu.2025.1461535

**Published:** 2025-01-29

**Authors:** Khalil Khalaf, Marc Chamieh, Natalia Welc, Chandpreet Singh, Joanne Lynn Kaouk, Aiden Kaouk, Andrzej Mackiewicz, Mariusz Kaczmarek, Bartlomiej Perek

**Affiliations:** ^1^ Doctoral School, Poznan University of Medical Sciences, Poznan, Poland; ^2^ Department of Cancer Immunology, Poznan University of Medical Sciences, Poznań, Poland; ^3^ Department of Cardiac Surgery and Transplantology, Poznan University of Medical Sciences, Poznan, Poland; ^4^ Department of Spine Disorders and Pediatric Orthopedics, Poznan University of Medical Sciences, Poznań, Poland; ^5^ Department of Dermatology, Poznan University of Medical Sciences, Poznan, Poland; ^6^ Department of Internal Medicine, University of California, Los Angeles (UCLA) - Kern Medical Center, Bakersfield, CA, United States; ^7^ Department of Science, Louisiana State University, Lousiana, LA, United States; ^8^ Department of Natural Sciences, The University of Texas at Austin, Texas, TX, United States; ^9^ Department of Cancer Diagnostics and Immunology, Greater Poland Cancer Center, Poznań, Poland

**Keywords:** cardiovascular disease, interferon, monocytes, lymphocytes, interleukin, atherosclerosis, plaque, chemokine

## Abstract

Atherosclerosis, previously regarded as a lipid storage disease, has now been classified as a chronic inflammatory disease. The hardening of arterial vessels characterizes atherosclerosis due to the accumulation of lipids in the arterial walls, eliciting an inflammatory response. The development of atherosclerosis occurs in various stages and is facilitated by many clinical factors, such as hypertension, hyperlipidemia, and inflammatory status. A large arsenal of cells has been implicated in its development. This review will summarize the phases of atherosclerotic formation and all the cells involved in either promoting or inhibiting its development.

## 1 Introduction

The development of atherosclerosis was previously thought of as a fatty storage disease affecting more modern populations due to diet and lifestyle. With our current knowledge and the multifactorial facets of atherosclerosis, it has been present longer. Although the past prevalence cannot be determined, a study was conducted in which the CT scan analysis of 137 mummies from various geographies was performed. This study yielded concrete presence of atherosclerosis in 34% of samples (1).

As the world’s leading cause of death, cardiovascular diseases (CVDs) affect both blood vessels and the heart. CVD can be caused by a variety of factors, such as lifestyle, obesity, smoking, socioeconomic status, diabetes, and so on. As of 2021, the World Heart Federation reported that over half a billion individuals are affected by CVD, with 20.5 million deaths related to CVD, representing 1/3 of worldwide deaths. Although new treatment protocols and lifestyle modifications have been shown to improve the prognosis of patients suffering from CVD (2), this number is approximately 60% higher when compared to the previously recorded deaths related to CVD 35 years ago. With all the current medical advancements, the following high number is primarily due to the growing population and aging (2). Atherosclerosis, a word derived from Greek roots, can be broken down into “atherosis,” which corresponds to fat accumulation and macrophages, and “sclerosis,” indicating fibrous tissue consisting of connective tissue, smooth muscle cells, and leukocytes. In the early 19th century, Jean Lobstein introduced the term atherosclerosis, bringing more profound meaning and understanding to arterial conditions (3). A few years later, two pioneers in their field proposed contradicting theories in the development of atherosclerosis.

On the one hand, Austrian physician Carl Von Rokitansky proposed a hypothesis of atherosclerosis development in a “thrombogenic” theory. He speculated that vascular damage from mechanical sources or others was responsible for atherosclerotic plaque formation (4). On the other hand, German physician Rudolf Virchow hypothesized that various clusters of immune pro-inflammatory cells already present within the vessels were responsible for the development of atherosclerosis (5). It wasn’t until the late ‘90s that Russell Ross proposed chronic inflammation following injury led to a cascade of events to form atherosclerotic plaques (6, 7). Human samples retrieved from Carl Von Rokitansky’s research showed the presence of T lymphocytes in early lesions, thus concretizing the importance chronic inflammation has on the development of atherosclerosis (3). As previously mentioned, the development of atherosclerosis is multifaceted, and we do not know why the formation and progression of atherosclerosis are accompanied by vascular and endothelial instability and immune cell overactivation. Still, at the center of it all is a chronic inflammatory process.

This review paper will discuss the stages of atherosclerotic development, the immune cells, and the immune mediators involved in its development.

## 2 The developmental process of atherosclerosis

As previously described, atherosclerosis develops in a multi-step process. This intricate course of action begins with the creation of fatty streaks, followed by the damaging of tissue then atheroma development and finally a more mature plaque formation (8).

### 2.1 Fatty streak development

In normal conditions, the low-density lipoprotein (LDL) concentration in both intracellular and plasma is proportionally balanced. When this “balance” is disturbed, LDL enters the endothelial space through endocytosis. Due to the high concentration of proteoglycans within the intimal layer and their high affinity, lipoprotein accumulation is facilitated (8). High affinity of LDL to proteoglycans, leads to their disproportionate accumulation and triggers oxidation of LDL particles by reactive oxygen species (ROS) (i.e., mitochondrial myeloperoxidase- MPO, xanthine oxidase- XO, lipoxygenase- LOX) to generate oxidized form (ox-LDL) (9–11). Ox-LDL and apolipoprotein B (ApoB) serve as the ligands for scavenger receptors (SR-AI/II; CD36; and SR-BI) and facilitate the entry of macrophages responsible for clearing these excess molecules in the intimal layer (12). It is believed that specific epitope oxidization on ox-LDL triggers both humoral and innate immunity to facilitate macrophage activity (13). In addition to forming ox-LDL, ROS activity byproducts such as lysophosphatidylcholine (LPC) induce an inflammatory response (14). In addition, MPO ox-LDL increased the inflammatory response by reducing IL-10 activity in macrophages (15).

Furthermore, the inflammatory process increases the activation of endothelial cells (EC) and smooth muscle cells (SMC) by enhancing the expression of leukocyte adhesion molecules VCAM-I and ICAM-I to increase the attraction of macrophages and lymphocytes (14). While macrophages internalize ox-LDL to form foam cells, they also act as antigen to T cells, further enhancing the release of pro-inflammatory cytokines (14). This iterative pattern of cytokine released by macrophages and lymphocytes will lead to the recruitment of more macrophages, which will internalize more ox-LDL and form foam cells. As previously stated, the function of macrophages is to internalize ox-LDL. Some will be returned to circulation, some foam cells within the intimal layer will eventually die off by apoptosis. An increased ratio of entering LDL versus exiting, the propensity for foam cells to reside in the intimal layer increases and thus results in streak formation (16).

## 3 Atheroma formation

Due to vascular tissue damage, SMC and EC secrete pro-inflammatory cytokines (i.e., TNF-α, IL-1) (17). The tissue destruction causes the migration of SMC to the lumen. This migration is accompanied by the synthesis of extracellular matrix (ECM) and forming a fibrous cap over the damaged area (18). The latter one houses dense collagen, T lymphocytes, macrophages, and SMC, resulting in a fibrous bulging structure forming a mature atherosclerotic plaque and reducing blood flow (19). As a method to correct this anomaly, macrophages and T cells will gather in the periphery, T cells will increase the secretion of TNF-α to prevent SMC secretion of collagen (20), and macrophages will increase the secretion of metalloproteinases (i.e., Matrix metalloproteinases – MMPs; and A-Disintegrin and metalloproteinases – ADAMs) to lyse the ECM (21). The “corrective” attempts by macrophages and T cells can destabilize the fibrous structure, expose lipid contents and collagen to blood products that facilitate platelet aggregation and eventually clot formation (16, 22).

## 4 Multi-cellular immune involvement in the process of coronary artery disease

**Figure 1:**
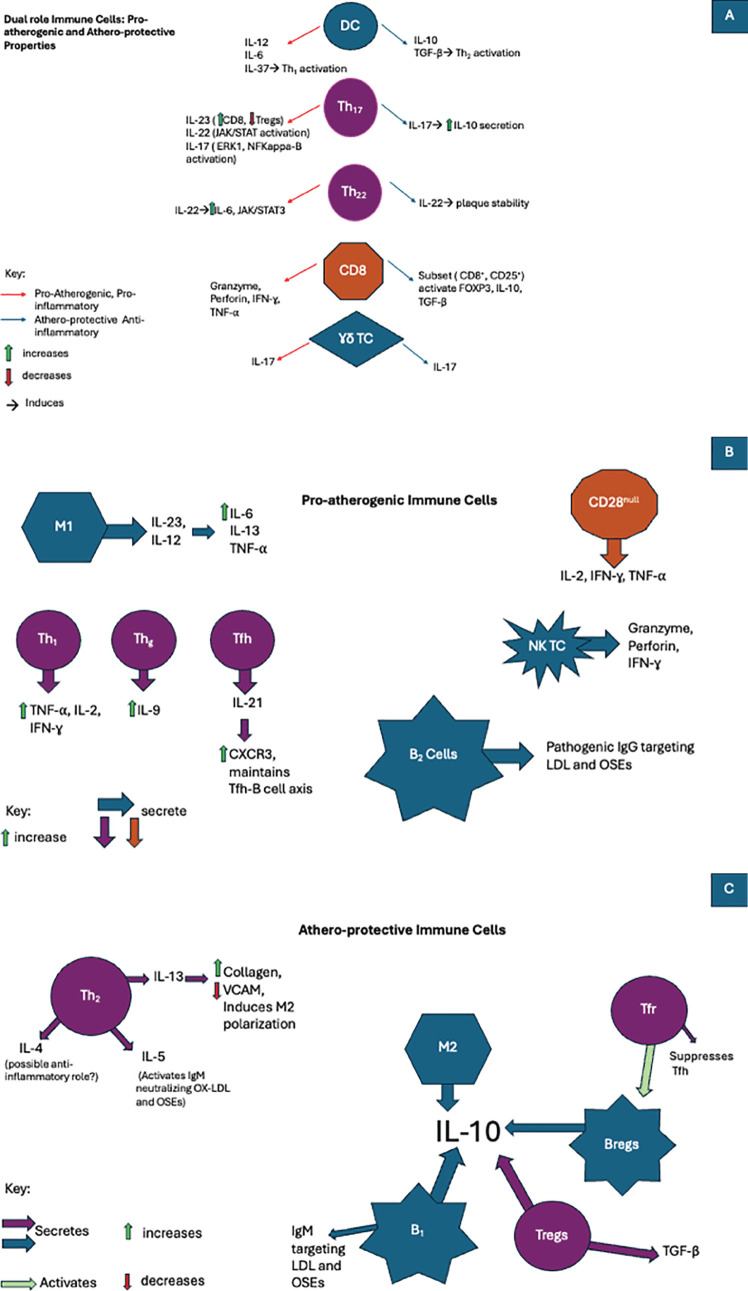
In atherosclerosis immune cells are involved in the development of pro-inflammatory, anti-inflammatory, or even both pathways when exposed to various cytokines. **(A)**The duality of the immune cell response in atherosclerosis: Pro-atherogenic and artheroprotective activity. **(B)** The role of pro-atherogenic immune cells in atherosclerosis. **(C)** The role of athero-protecrtive immune cells in atherosclerosis.

It was previously believed that macrophages were the sole immune cells subtype within arteriosclerotic arteries, but the early 80s defined a critical moment as the presence of other leukocytes was established (23). Various studies have confirmed the presence of standard immune cells in the atherosclerotic areas of arteries (7, 24). The level of inflammation and degree of vessel damage determine a number of recruited immune cells. This recruitment, in turn, acts upon a feedback loop and is dependent upon a certain balance between entering-vs-exiting cells and apoptosis-vs-survival of them. In this section, we will elaborate on the presence of various immune cells encountered in atherosclerosis and explain their contribution to its development (see **Figure 1**).

### 4.1 Monocytes and their subsets

In the past, monocytes were considered to be circulating immature precursors of terminally differentiated macrophages and dendritic cells (DCs) in the tissue (25). A study conducted on mice has shown the presence of them in areas unaffected by atherosclerosis but labeled as atherosclerosis-prone regions (26). In the consecutive years, more evidences have been collected the majority of resident macrophages and DCs did not derived from circulating monocytes (27, 28). However, in the inflamed tissues more monocyte-derived cells (both macrophages and EDCs) are noted but they present different biological properties from resident ones (25).

Circulating monocytes consist of three major distinct functionally (even before reaching the tissues) subpopulations that can be distinguished from each other by the expression of the surface markers such as CD14 and CD16 (29). Classical monocytes (CD14+CD16−) display phagocytosis and ROS production potential, manifest strong affinity to the injured or inflamed tissue (related to surface CD14 expression) and possess the ability to differentiate into monocyte-derived tissue macrophages and DCs (30, 31). Of note, they produce the relative small amounts of cytokines (attributable to CD16 expression) (32). Therefore, they play a crucial role in the local inflammation and its resolution in tissues (33). Intermediate monocytes (CD14+CD16+) are well-suited for antigen presentation (high class II molecule expression), cytokine secretion, apoptosis regulation, and differentiation (34). They present ROS production and phagocytosis potential comparable to classical subpopulation (29). The non-classical monocytes (CD14dimCD16+) are involved in complement and Fc gamma-mediated phagocytosis (clearance of dying cells) as well as neutrophil adhesion through secretion of TNF-α (29, 35).

Application of mass cytometry technology revealed that even aforementioned subpopulations are not homogenous and further subdivisions were proposed (36). Briefly, distinct levels of IgE, CD61/CD9 or CD93/CD11a expressions enabled identification of 4 subsets of classical monocytes whereas differences in CD9 and 6-sulfo LacNAc (SLAN) 3 subtypes of non-classical ones (36, 37). Variable expression of these receptors may affect substantially biological properties and eventually clinical significance (see below). For example, CD14dimCD16+SLAN+ cells exhibited increased efferocytosis than SLAN− non-classical monocytes (36).

In the case of inflammation, monocyte recruitment occurs by increased expression of P-selectin on the endothelial surface (38). It has been shown that a lower expression of P-selectin was linked to less monocytes accumulation and decreased fatty streak formation (39). E-selectin was shown to overlap with P-selectin to provide better support for leukocyte rolling to the site of inflammation (39). In addition, with the assistance of surface chemokines (i.e., CXCL4, CXCL2, CCL5, and many others) serving as homing signals and activation molecules for monocytes, vascular adhesion molecules (VCAM-1) were shown to be critical in the vascular rolling process of leukocytes to the inflammatory sites (40). Monocytes recruited to inflammatory sites differentiate into dendritic cells (DCs) or macrophages (41). The selection process of either one needs to be better established as they both work in tandem to reduce the devastating processes of atherosclerotic insult.

Clinically, a detailed assessment of subpopulations’ counts instead of total monocyte in the peripheral blood samples can provide additional value in risk stratification of atherosclerosis development and/or its complication prevalence. Increased counts of the classical monocytes have been shown to predict future cardiovascular risk independently of other risk factors (42). In the diabetic patients [typically characterized by increased monocytopoesis (43)] with advanced coronary artery disease, not only absolute number of classical monocytes but also relative increase at the expense of both intermediate and non-classical subset was noted (44). The authors speculated that this observation followed a global increase in monocyte production by the bone marrow and simultaneous promoted adhesion to the arterial endothelium of intermediate and nonclassical monocytes to the endothelium of atherosclerotic arteries (45). A population of the SLAN+ non-classical monocytes correlates with the coronary arteries Gensini score, a marker of atherosclerosis severity (36). These cells contribute, as mentioned before, to efferocytosis, a process of paramount importance in preventing the progression of atherosclerosis through maintenance of vascular homeostasis (46). Therefore, it seems that in the early stages of the atherosclerotic plaques formation an increased classical monocyte counts dominates and it could be considered a predictive marker, while later on, the presence of necrotic cores may rather recruit non-classical monocytes to control vascular homeostasis and the clearance of debris (47, 48).

#### 4.1.1 Macrophages

Macrophages are the first and most abundant immune cells in atherogenic areas (49). Likewise to monocytes, cellular plasticity and heterogeneity are also inherent features of macrophages (50). A single-cell RNA-seq analysis of aortic atherosclerotic plaques provided an insight into their heterogeneity and enabled to identify following three main subsets of macrophages: (I) inflammatory (II) resident-like and (III) macrophages with a high expression of Trem2 and genes associated with lipid-metabolic pathways and cholesterol efflux (51, 52). In the recent study, a dualistic nature of cells with Trem2 expression wa described. In early plaques, they promote lipid uptake and lesion growth, whereas in advanced plaques they enable macrophages survival and better stability of the atherosclerotic plaques (53).

As innate cells, macrophages hold a multitude of functions such as the intake of excess lipids, producing various pro-inflammatory cytokines, and expressing multiple pattern recognition receptors (PRRs), including toll-like and scavenger ones (TLRs and SRs, respectively) to activate both innate and adaptive immune cells. SRs are critical for recognizing and internalizing ox-LDL destined for lysosomal degradation. With all the advancements in atherosclerosis development, SRs present as a double-edged sword. On the one hand, they has been shown to contribute to protecting arteries from atherosclerosis development but on the other, various SRs may facilitate atherosclerosis by attracting more macrophages and contributing constant inflammation in the region (49).

It is well known that undifferentiated macrophages can polarize into classically activated (M1) and alternatively activated (M2) macrophages under different microenvironmental conditions (54). A direction of their polarization depends on exposure to such cytokines as interleukins, interferon but also lipopolysaccharide or noncoding RNAs. M1 polarized macrophage occurs in a milieu high in IFN-γ and TNF, while M2 polarization in a milieu high in IL-13 and IL-4 (55). Generally, the classic M1 phenotype comprises high levels of IL-23 and IL-12, while M2 one high expression of IL-10. M1 produces pro-inflammatory cytokines (IL-6, IL-1-β, and TNF) and effector molecules (i.e., ROS) whereas M2 large amounts of anti-inflammatory cytokines (IL-10) that alleviate inflammation and promote tissue repair (50). Both polarized versions are present within the atherosclerotic region in varying degrees. The level of inflammation determines whether the atherogenic process will be inhibited or promoted. The increased apoptosis of foam cells has been linked to anti-atherogenicity (55). In contrast, resistance or impairment to apoptosis has been shown to be associated with a higher intensity of inflammation, in a consequence secondary necrosis of other cells within the plaque (ECs, SMCs, and leukocytes) and proatherogenic process (56).

A role of macrophages in atherosclerotic plaque biological history depends on its formation stage. At the beginning, monocytes are recruited to form lesion then they, as monocyte-derived macrophages, promote its growth followed by local expansion (57). Later, their proliferation is the predominant contributor of expansion and the main source of the foam cells in the plaques (58). ScRNA-seq provided a more detailed transcriptional landscape of macrophages in atherosclerosis, that challenged the traditional view (59). In the high-fat diet (HFD) mice model was disclosed that Trem2hi dominated in the control healthy mice while the inflammatory and resident-like macrophages promoted the progression of atherosclerosis plaques (59).

Moreover, these macrophages may also actively participate in the calcification process of the atherosclortic plaques’ caps (60). In addition to CD14+, they were shown to express alkaline phosphatase and osteocalcin involved in development of the calcium deposits (61).

#### 4.1.2 Dendritic cells

DCs, like macrophages, were noted within the intimal layer in atherosclerotic-prone regions of aorta that are exposed to high shear‐stress blood flow (62). In the atherosclerotic arterial walls, they are well represented in both the plaques and in the adventitia (63). It is likely that during development of atherosclerosis, DCs are recruited into theses region at least partially due to the physical force of blood flow.

Primary, at least for subpopulations of the DCs have been identified in the aortic wall (64). Two subpopulations of the conventional DCs such as CD11c+CD11b− and CD11c+CD11b+ (an abundant form; 50-60%) in the normal intima (65). Notable, both were suggested to play possible anti-atherogenic role (64, 66). The third DC subset [CD45RA+ plasmacytoid DCs (pDCs)], primarily noted in the adventitia, was found to promote atherosclerosis development through secretion large amounts of the type 1 interferons IFN-α and IFN-β (67). The forth, atherogenic C-C motif chemokine ligand 17 (CCL17+) DCs, were reported only in the intima and adevntitia of atheroscerotic arteries (68).

Recently, applying modern approaches as single-cell RNA sequencing and mass cytometry (high-dimensional cytometry by time of flight; CyTOF), four DCs subsets have been identified and classified as cDC1, cDC2/moDC (monocytes-derived), pDC and mature DCs (69). They differed between each other with expressions of the numbers of receptors. Importantly, during progression of atherosclerosis, only pDCs expression was increased but the others remained reduced (cDC2) or unchanged (cDC1) (70).

DCs were shown to produce a mesh-like network whose interactions with other cells varied in pre- versus post-atherosclerotic lesions and thus were considered to maintain homeostasis (62). Although their function in healthy vessels has not been well established, much better their role in atherosclerotic has been described (69). Analyzing the associations of different subset of DCs with the development of atherosclerosis, the actions of DCs in the disease promotion were divided into direct and indirect. The earlier includes antigen presentation (APC) to stimulated adaptive immunity by means of nearby T cell activation, efferocytosis of cholesterol-rich apoptotic cells, internalization of ox-LDL with formation of foam cells following macropinocytosis, receeptor-mediated endoctosis and direct uptake of lipoproteins from circulation (64, 71–73). The indirect roles involves production of many cytokines either pro-inflammatory (IL-12, IL-6, IL-37) or anti-inflammatory (IL-10, TGF-β) as well as activation of Th1, Th2, Th17 (pro-inflammatory pathway) and Treg cells (anti-inflammatory route). They are also able to govern indirectly the polarization of macrophages (64).

### 4.2 Lymphocytes and their subsets

In healthy tissue, T lymphocytes were found to reside within the adventitial layer. Their movement to the site of inflammation is triggered by exposure to lipid deposition and damage within endothelial cells (74). As per other immune cells, lymphocyte infiltration is triggered by chemokine receptors (CCRs and CXCRs) binding to their respective ligands (74). Flow cytometry analysis within atherosclerotic plaques confirmed the predominance of T cells within the lesion (75). To initiate the activation of T cells, they must first come in contact with specialized proteins presenting processed peptides resulting from phagocytosis, mainly MHC-I and MHC-II (Major histocompatibility complex) (76). MHC-I is present in almost all nucleated cells and is crucial in activating CD8+ cells (cytotoxic T lymphocytes). At the same time, MHC-II is mainly found in APCs and serves to activate CD4+ cells (helper T lymphocytes). In addition to their vital role in immune activation, these molecules are highly polymorphic, allowing them to present a wide range of processed antigens (77). In a hyperlipidemic mouse model, the importance of MHC-II in atherosclerosis was depicted, as mice deficient in this protein had a twofold increase in CD8 cells while no CD4 activation, thus resulting in higher proatherogenic development (78). An arsenal of various lymphocytes was seen to be present whether to promote or inhibit the further development of atherosclerosis. Their role depends upon the inflammation level and the presence of various other immune cells and mediators released.

#### 4.2.1 CD4 T cells

As central players in adaptive immunity, CD4 cells (T helper cells) enhance CD8 response, assist B cells in antigen generation, and regulate the activity of macrophages (79). Various subsets of Th cells within atherosclerotic plaques have been described in the literature. After Th cell progenitors develop in the bone marrow, they migrate to the thymus, and undergo thymic education (80). During this stage, random generations of T cell receptors (TCR) are generated to form unique receptors on T cells (81). A process of selection follows the latter. Positively selected naïve cells develop into Th0 (CD4+/CD8-), while negatively selected cells undergo apoptosis (82). After their release from the thymus into the blood and lymphatic circulation, Th subset polarization varies depending on the cytokines and antigens presented by APCs (83).

##### 4.2.1.1 Th1 cell subtype

Th1 cells were shown to be the predominant subtype of CD4 in both human and mouse atherosclerotic plaques (84). In the presence of IL-18, IFN-γ, and IL-12, once APCs interact with naïve Th cells, a characteristic transcription factor TBX-21, also known as T-bet, is upregulated to promote Th1 subset differentiation (85). Once fully activated, Th1 will induce production of pro-inflammatory cytokines such as TNF, IL-2, and IFN-γ (83). The importance and proatherogenic function of Th1 was depicted in a study that showed that hyperlipidemic mice deficient in Th1 had reduced atherosclerotic development (86). The progression of atherosclerosis has been linked to the high concentration of ox-LDL and ApoB. As previously stated, Th1 differentiation yields high amounts of pro-inflammatory cytokine IFN-γ (87). The importance of inflammation and the proatherogenic effects of Th1 was further shown in a study in which mice models with deficient cholesterol concentrations were injected with IFN-γ, and atherosclerotic lesion growth two-fold compared to the control group (88).

##### 4.2.1.2 Th2 cell subtype

After Th1, Th2 cells represent the second most abundant cell subtype within atherosclerotic plaques (84). The differentiation of thymocytes into Th2 cells is triggered by excess amounts of IL-4, which triggers the expression of transcription factor GATA-3 (89). In addition to aiding in promoting B cell response, this subset is characterized by the production of IL-13, IL-4, and IL-5. During a 15-year follow-up analysis of blood drawn in patients with coronary artery disease (CAD), the accumulation of Th2 cells was shown to possess atheroprotective properties. It decreased cardiovascular disease (CVD) development in women (90). In experimental models, IL-5 was shown to induce the activation of adaptive immunity with IgM epitopes specific to ox-LDL, inhibiting atherosclerosis progression. In contrast, in IL-5 deficient models, atherosclerosis progression was prominent (91).

Furthermore, IL-13 has shown its atheroprotective role by increasing the collagen content within established atherosclerotic lesions, decreasing the expression of VCAM-1 responsible for attracting more macrophages to the site, and inducing the polarization of macrophages into the M2 phenotype (92). The most abundantly produced, IL-4, with its inhibitory actions against Th1, is considered anti-inflammatory, but in the context of atherosclerosis, its role remains controversial. On the one hand, animal model studies have shown that despite elevated levels of serum lipids, IL-4 deficiency reduced the incidence of atherosclerosis development (93). Other studies supported the following: in IL-4 knockout mice, atherogenesis was inhibited despite their hyperlipidemia; the authors proposed the importance of IL-4 to be more prominent in the early phases of atherogenesis (94). Other animal studies have also reported that IL-4 has no direct effects on the development of the lesion (95). In contrast, some studies show the atheroprotective functions of IL-4 in their ability to induce M2 polarization (96). All these controversial results indicate that IL-4 is part of an intricate system within the atherosclerotic process and should be further investigated.

##### 4.2.1.3 Th17 cell subtype

Th17 differentiation is achieved under TGF-β and IL-6, the lineage-defining transcription factor ROR-γ-t (Retinoic acid-related orphan receptor ROR-γ-t) (97). The presence of Th17 cells within atherosclerotic plaques remains controversial. Depending on the animal model used, these cells appear to possess dual actions affecting the development of atherosclerosis. Their proatherogenic properties rely upon their capacity to induce a proinflammatory environment by release of GM-CSF, IL-6, and IFN-γ (98). Their activation relies upon preexisting cytokines such as IL-23 and IL-17, also part of their cytokine arsenal (99). Th17 polarization occurs via different pathways. The indirect pathway of polarization occurs by reversion after Tregs (regulatory T cells) lose their immunosuppressive effects (i.e., loss of FOXP3 expression) (100). The direct pathway involves the presence of a multitude of proinflammatory cytokines (i.e., IFN-γ, TNF-α, Nuclear Factor kappa B (NF-KB), GM-CSF) (101). The Th17 cytokine arsenal mainly comprises of IL-23, IL-17, and IL-22 (102–104). IL-17 has been associated with both atherogenic and atheroprotective actions.

On the one hand, they induce the activation of various proinflammatory signaling pathways, such as ERK-1 and NF-kappa-B, producing TNF-α, IL-8, IL-6, and IFN-γ (105). Conversely, a subtype of IL-17 activated by TGF-β and IL-6 has been shown to produce IL-10, inhibiting adhesion molecules on VSMC and DCs, thus preventing their proinflammatory destructive action (106, 107). IL-23 was shown to possess proatherogenic properties by inducing the polarization of Th17 by inhibiting FOXP3. As a result, the high expression of IL-23 was a direct cause of the imbalance between CD8 cells and Tregs. It should also be noted that they are also expressed in proinflammatory cells such as DCs and M1 macrophages (108). IL-22 is involved in many proatherogenic mechanisms; it has been shown to regulate vascular smooth muscle cell migration, angiogenesis, and the activation of proinflammatory signaling pathways such as JAK/STAT (109). In addition, IL-22 within atherosclerosis was associated with the accumulation of cholesterol and attraction of M1 macrophages, resulting in the release of MMP-9 and various proinflammatory cytokines (110, 111).

##### 4.2.1.4 Th9 cell subset

As the primary source of IL-9, Th9 was previously considered a subset of Th2 cells. The stimulation of IL-9 production is primarily dependent upon IL-4 and TGF-β (112). A comparison of blood plasma between healthy and CAD patients showed an increase in IL-9 without notable differences in Th9 concentration (113). In a study comparing healthy mice and atherosclerotic mice models, the elevation of Th9 cells and IL-9 concentration directly correlated with the level of ox-LDL and inflammation in atherosclerotic lesions (114). In addition, treating atherosclerotic mice with anti-IL-9 receptor antibodies significantly diminished the progression of atherosclerosis by reducing inflammatory cytokines (114, 115).

##### 4.2.1.5 Th22 subset

Much like Th17, Th22 requires the action of ROR-γ-t as a positive regulator for their differentiation, while transcription factor T-bet acts as a negative regulator (116, 117). As a primary source of IL-22, Th22 cells have been observed in various autoimmune illnesses and chronic inflammatory processes such as atherosclerosis. A study conducted to compare the effects of atherosclerosis on ApoE deficient mice vs. healthy mice has shown that the presence of Th22 and IL-22 was directly associated with the level of inflammation (118). Like many other interleukins, IL-22 derived from Th22 cells has been proven to function dually. On the one hand, IL-22 proved to be proatherogenic by activation of crucial inflammatory signaling pathway IL6/JAK2/STAT3 (119), which in turn attracted more inflammatory cells, facilitated the differentiation of Th17, and enabled SMC migration and modification into a less flexible phenotype, thus worsening the progression of atherosclerosis (117, 118). On the other hand, the action of IL-22 was shown to induce plaque stability and was shown to repress the proatherogenic metabolites (trimethylamine N-oxide and lipopolysaccharide) released by gut microbiotas (120).

##### 4.2.1.6 Regulatory T cells subtype

Generated within the thymus, Tregs serve as guardians to maintain tolerance and inhibit immune overactivation of both innate and adaptive cells by secreting signaling molecules such as TGF-β and IL-10 (121). The defining attribute of Tregs is the expression of FOXP3, but the following was bona fide in mice as it was seen to appear in maturing human CD4 cells transiently. The important atheroprotective properties of FOXP3-Tregs were extensively documented in atherosclerotic mice models (122). Similarly, in human patients, the low concentration of Tregs and their respective atheroprotective cytokines was directly associated with atherosclerosis progression and its severity (123, 124). Additionally, a large cohort study confirmed the importance of Tregs, as their low concentration directly correlates with plaque instability and increased risk of myocardial infarction (125). In mice models of atherosclerosis, elevated levels of IL-10 were associated with a decrease in proatherogenic cellular infiltration and activation (126).

In contrast, mice deficient in IL-10 were reported to present with plaque progression in a proinflammatory-dependent manner (127). In atherosclerotic mice models, TGF-β was seen to possess plaque-stabilizing properties (128). Its anti-inflammatory effects also reside in its indirect ability to maintain the atheroprotective activity of Tregs and its direct suppressive activity of both innate and adaptive cells, such as natural killer cells (NK), DCs, M1 macrophages, and effector T cells (129, 130). Their importance as immune regulators was also expressed in the literature, as was their plasticity (131). The loss of FOXP3 activity was associated with a diminished ability to regulate immune overactivation and sometimes their conversion to a proatherogenic variant follicular helper cells (Tfh) (131).

T regulatory cells present in various compartments of the body constitute a heterogeneous population of lymphocytes exhibiting a significant potential for immunophenotypic plasticity. Treg cells present in the thymus, known as thymus-derived (tTreg) or more commonly natural Treg cells (nTreg), arise as a separate developmental lineage. However, Treg also arise outside the thymus in tissue locations, which is why they are called induced; iTreg, adoptive; aTreg or periphery derived; pTreg). It has been shown that approximately 80% of Treg cells originate from the thymus, while the remaining 20% differentiate from conventional T cells present in the periphery. Both tTreg and pTreg cells reside primarily in lymph nodes, but are also present in peripheral blood, where they constitute a population of 10-15% among T cells CD4+ (132). For all T reg, the hallmark is the expression of the transcription factor FoxP3+, therefore, in order to distinguish tTreg from pTreg cells, Thornton AM et al. proposed to assess the expression of the transcription factor Helios (133). Treg activity is crucial for maintaining both central and peripheral immune tolerance, which protects the organism from autoaggression. Therefore, the immunophenotypic plasticity of Tregs is their feature that allows functional adaptation of these cells to changing conditions in the surrounding microenvironment. Through TGFβ, activated tTregs provide a boost to naive T cells that adopt the regulatory phenotype pTregs. In patients with subclinical cardiovascular disease (sCVD), APOB-specific CD4+/FoxP3+ T cells were found to express transcription factors characteristic of other T helper cell subpopulations, such as ROR-γt (Th17) and T-bet (Th1). This expression profile suggests that these cells lose their regulatory function and simultaneously adopt a proinflammatory phenotype (134). Some sCVD patients have APOB-specific Tet+ T cells. This first scRNA-Seq study of previously unknown cells showed that Tet+ cells are not true Tregs, although they share some genes with the transcriptome of Tregs, which is why the authors proposed to call them Treg-like. A transcriptomic study of human APOB-specific CD4 T cells suggests that the transformation of Tregs into exTregs promotes the development of atherosclerosis in both mice and humans. Freuchet A. et al. found that Tet+ cells initially resembling Tregs in sCVD patients become exTregs and acquire a phenotype similar to Tmemory cells (135) The authors showed that exTreg cells lose expression of the FoxP3 factor, but similarly to NK cells, they gain expression of the CD16 and CD56 antigens. In addition, most exTreg cells synthesize and secrete IFN-γ, and also express granzyme B and proinflammatory chemokines CCL3, CCL4, and CCL5. This proinflammatory activity of exTreg cells induces the recruitment of monocytes, some of which can differentiate into macrophages, which promotes the exacerbation of atherosclerosis. The authors therefore postulate that the conversion of Treg cells to exTreg cells may be part of the mechanism associated with the disruption of tolerance to autoantigens observed in atherosclerosis.

##### 4.2.1.7 Follicular helper T cells subtype

As necessary components enabling isotype antibody switching within germinal centers (136), Tfh cell frequency has also been associated with their pro-inflammatory contributions in autoimmune diseases (137). In proatherogenic conditions, Tfh cells were shown to increase the expression of proinflammatory chemokine CXCR3, which is vital for CD8 cell migration (138). The accumulation of Tfh cells and overactivation of cells in germinal centers was associated with the worsening of atherosclerotic development. The disruption of the Tfh-B-cell axis by Tregs was shown to result from the interruption of ICOS-ICOSL (T cell costimulatory and its ligand), thus diminishing the progression of atherosclerosis (139). The most abundantly produced cytokine by Tfh cells is IL-21, which has been crucial for maintaining the Tfh-B-cell axis. Inhibition of IL-21 was directly associated with diminished progression of atherosclerosis (140).

##### 4.2.1.8 Follicular helper regulatory T cells subtype

Another regulatory cell subtype is Tfr. It was shown to be a critical player in providing aid within germinal center activity and adequate differentiation (141). The atheroprotective actions of Tfr affect the Tfh-B-cell axis both directly and indirectly (141). In the direct approach, in response to ApoE-deficient mice fed with a high-cholesterol diet, the differentiation of Tfr was significantly increased, suppressing Tfh activity (142). In addition to suppressing proinflammatory cells, the indirect activity of Tfr relies upon the activation of regulatory B cells (Bregs), which in turn inhibit the B-cell axis within germinal centers (142).

##### 4.2.1.9 CD28 null T cell subtype

As an essential signal transducer, CD28 serves as a membrane receptor required to recognize surface molecules of the B7 family (i.e., CD80) (143). Adequate responses to activate naïve lymphocytes depend on interaction with APCs and the B7 family molecules (144). Unlike their Th cell counterpart, CD4+ and CD28+, CD28 is crucial for differentiating naïve CD4+ cells into Th cells. Still, their absence has been linked to promoting an inflammatory environment (145). CD28 null cell types are terminally differentiated, and their proinflammatory status is associated with producing various proinflammatory cytokines (i.e., IL-2, IFN-γ, and TNF-α) (146). Furthermore, studies have shown that *in vitro* this subtype possesses some cytotoxic activity towards endothelial cells due to their ability to produce enzymes specific to CD8+ and NK cells (mainly granzyme a/b, perforins) (147). The increased presence of this cellular subtype is directly correlated with the severity of arterial disease (148, 149).

#### 4.2.2 Other lymphocytes and their role in atherosclerosis

##### 4.2.2.1 CD8+ T cells

After recognition of processed antigens on MHC-1, CD8 cells become active, and clonal expansion occurs to form cytotoxic lymphocytes (Tc) (150). Tc is well known for providing a pro-inflammatory milieu by releasing various inflammatory enzymes to rid the body of foreign elements and cancer cells (150). In both mice and humans, the ratio of CD8 to CD4 is elevated depending on the level of atherosclerosis. They were seen to be widely present within the fibrous cap (151, 152). T cell receptor sequencing studies within advanced atherosclerotic plaque revealed that Tc expanded rapidly, suggesting that clonal expansion occurred within the fibrous cap (153). Although having a reputation for providing a pro-inflammatory milieu, Tc exhibits both athero-protective and pro-atherogenic properties. In ApoE and Tc deficient mice models of atherosclerosis, no changes were observed in the progression of atherosclerosis (154).

In contrast, other studies have shown that in ApoE-deficient mice, the depletion of Tc correlated with the regression of atherosclerosis (155). When comparing atherosclerotic mice models to a control group, Tc in atherosclerotic mice produced more proinflammatory elements such as granzymes and IFN-γ, thus contributing to lesion progression and core necrosis. The following was suggested to result from granzyme-mediated cell death of various cells (endothelial cells, macrophages, and SMC), leading to core necrosis (156). Subsequent studies focused on advanced-stage atherosclerosis have shown that antibody-dependent depletion of Tc was associated with worsening of plaque stability due to the increased influx of macrophages and Th1 cells (157, 158). To further study the effects of cytokines released by Tc, adoptive transfer of modified Tc deficient in granzymes, IFN-γ, and TNF-α to lymphocyte depleted, ApoE deficient mice proved not to affect the worsening progression of atherosclerosis (155). The atheroprotective role of Tc belongs to a specific subset within the Tc family. These regulatory Tc have been documented within atherosclerotic plaques and were shown to express both CD8 and CD25 phenotypically (159, 160). To further evaluate their function, adoptive transfer of this cellular subtype in apoE-deficient mice diminished the presence of CD4 and Tc within the plaque. It slowed the progression of atherosclerosis plaque formation (161). Various studies have explained the immunosuppressive activity that shows how similar these cells are to Tregs, with the high expression of immune modulators such as FOXP3, IL-10, and TGF-β (162).

##### 4.2.2.2 γ-δ T cells

As previously mentioned, T cells develop within the thymus, with two distinct subtypes, the majority being α-β T cells and a minority being γ-δ T cells (163). The two mentioned cell types vary in the arrangement of their TCRs (T cell receptors) (163). On the one hand, α-β T cells require both positive and negative selection within the thymus, and their activation occurs within peripheral tissue after encountering MHCs presenting specific antigens. On the other hand, γ-δ T cells bypass the selection and become active within the thymus by recognizing processed proteins and non-protein products without MHCs (164, 165). Since their first discovery within atherosclerotic plaques in the 90s, more research is still needed to provide more information on this cellular subtype (166). In atherosclerosis, immune activation of γ-δ T cells directly correlates with their intracellular cholesterol content, indicating that cholesterol metabolism is critical for enhanced γ-δ TCR signaling and cellular proliferation and activation (167). Although their role in atherosclerosis has not been fully uncovered, these cells strengthen the progression of atherosclerosis by secreting large amounts of IL-17 (168). The proatherogenic function of IL-17 has been well documented in ApoE and LDL receptor (LDLR) deficient mice, whereas their atheroprotective role has only been observed in LDLR-deficient mice (169).

##### 4.2.2.3 Natural killer T cell subtype

The discovery of NK T cells within advanced atherosclerotic lesions has been documented in humans and mice (170). As innate lymphoid cells, they develop not only within the bone marrow but also the spleen, thymus, lymph nodes, and liver (171). Two subtypes of NK T cells have been uncovered: type I (invariant type), which possesses a few different TCRs, and type II, which presents a broader arsenal of TCRs (172). It should be noted that only the invariant type has been studied in atherosclerosis models. NK T cells undergo transition to their active forms in the presence of pro-inflammatory cytokines IL-15, IL-12, and IL-18 (173, 174). Experimental models suggest that NK T cells are proatherogenic in function (175, 176). Like cytotoxic lymphocytes, NK T cell’s pro-atherogenicity results from the pro-inflammatory secretion of granzymes, perforins and IFN-γ (177).

##### 4.2.2.4 B lymphocytes

B lymphocytes develop from hematopoietic stem cells within the bone marrow in specialized areas called “niches.” Their developments are cytokine-mediated, mainly IL-7, IL-4, IL-6, IFN, and so on (178). These cytokines target various genes to modulate cellular proliferation, differentiation, and B cell receptor (BCR) gene recombination to elicit diverse BCR (179). Unlike its T lymphocyte counterpart and other monocytes, B sales are the least represented within atherosclerotic plaques. However, their presence is abundant within adipose tissue (180). Murine models have been more extensively studied and have provided a broad division of cells like that found in humans. B Lymphocytes are divided into B1, B2 and regulatory B cells (Bregs).

B1 cells were seen to originate within the fetal liver and undergo regeneration within peripheral tissue. With their ability to travel to the bone marrow and spleen in a T cell-dependent manner, they predominantly produce IgM antibodies (181–184). B1 cells were deemed atheroprotective through the secretion of these antibodies, which target LDL, oxidative specific epitopes (OSEs), inhibiting the uptake of lipids by monocytes and eventually preventing the formation of additional foam cells as well as the secretion of pro-inflammatory cytokines (185, 186). Another atheroprotective mechanism lies in their ability to secrete large amounts of anti-inflammatory cytokine IL-10, which suppresses T-cell activation (187).

Also known as conventional B cells, B2 cells represent the vast majority. After being formed within the bone marrow, these cells mature in secondary lymphoid organs to finally form follicular and marginal B cells (184, 188). B2 cells have been described as pro-atherogenic by increased production of pathogenic IgG antibodies, which target OSEs and enable T cells to increase secretion of inflammatory cytokines such as IFN-γ (189). Furthermore, the depletion of B2 cells with B1 cells intact in mice models of atherosclerosis resulted in attenuation of inflammation and diminished progression of atherosclerosis (190).

Much like Tregs, regulatory B cells (Bregs) function to keep the immune system in check by secretion of inflammatory cytokine IL-10 (191–193).

## 5 Conclusion

The development of atherosclerosis occurs in progressive stages with fatty streak formation, atheroma development, and finally, atherosclerosis. Many factors facilitate its development, mainly hypercholesterolemia, lipid oxidation, hypertension and inflammation. It is now widely accepted that the development of atherosclerosis relies on pro-inflammatory conditions, thus labeling it as a chronic inflammatory disease driven by self-antigens (i.e., apolipoproteins, LDL). As detailed in this review, the multifaceted aspect of atherosclerotic plaque development is governed by various immune cells, some of which possess pro-atherogenic properties while others are athero-protective. Although our understanding of atherosclerosis has significantly advanced, more research is needed to target specific aspects of inflammation and prevent/treat or anticipate possible complications.
